# Synthesis of Pt–Pd Bimetallic Porous Nanostructures as Electrocatalysts for the Methanol Oxidation Reaction

**DOI:** 10.3390/nano8040208

**Published:** 2018-03-30

**Authors:** Yong Yang, Yanqin Cao, Lili Yang, Zhengren Huang, Nguyen Viet Long

**Affiliations:** 1 State Key Laboratory of High Performance Ceramics and Superfine Microstructure, Shanghai Institute of Ceramics, Chinese Academy of Sciences, 1295 Dingxi Road, Shanghai 200050, China; caoyq898@163.com (Y.C.); llyang@student.sic.ac.cn (L.Y.); zhrhuang@mail.sic.ac.cn (Z.H.); 2 Graduate University of Chinese Academy of Sciences, Beijing 100049, China; 3 Department of Electronics and Telecommunications, Saigon University, 273 An Duong Vuong Street, Ho Chi Minh 700000, Vietnam; nguyenvietlong@sgu.edu.vn

**Keywords:** Pt–Pd nanostructures, polyol process, electrocatalysts

## Abstract

Pt-based bimetallic nanostructures have attracted a great deal of attention due to their unique nanostructures and excellent catalytic properties. In this study, we prepared porous Pt–Pd nanoparticles using an efficient, one-pot co-reduction process without using any templates or toxic reactants. In this process, Pt–Pd nanoparticles with different nanostructures were obtained by adjusting the temperature and ratio of the two precursors; and their catalytic properties for the oxidation of methanol were studied. The porous Pt–Pd nanostructures showed better electrocatalytic activity for the oxidation of methanol with a higher current density (0.67 mA/cm^2^), compared with the commercial Pt/C catalyst (0.31 mA/cm^2^). This method provides one easy pathway to economically prepare different alloy nanostructures for various applications.

## 1. Introduction

Recently, direct methanol fuel cells (DMFCs) have been widely reported to exhibit high-energy conversion efficiency, a low operation temperature, and environmentally benign properties [1,2]. Consequently, they exhibit potential as an alternative to conventional combustion engines in next-generation mobile applications. Platinum (Pt) has been widely used as the highly-active catalyst in DMFCs and heterogeneous catalysis [3] because it possesses a unique intrinsic structure and is able to facilitate both hydrogen oxidation and oxygen reduction [4,5]. Unfortunately, the limited resource reserves and sky-high price of Pt has become one of the main barriers inhibiting the commercialization of DMFCs. Therefore, it would be beneficial to reduce the consumption of Pt by replacing it with non-noble metals while attempting to maintain the excellent properties previously mentioned.

To address this issue, various Pt-based bimetallic nanoparticles (Pt–Pd, Pt–Cu, Pt–Ni, Pt–Co etc.) have been reported to serve as economical and effective candidates for Pt catalysts in various fields [6,7,8]. Firstly, the introduction of traditional metal compositions acts to reduce the consumption of Pt catalysts [9,10,11]. Furthermore, the introduction of secondary metal elements can modify the crystallographic and electronic structures of Pt nanoparticles, and so, improve the binding energy between Pt and the poisonous species [9]. Among the various noble-metal nanomaterials available for catalytic applications, palladium (Pd) shares the same face-centered cubic structure and a nearly identical lattice with Pt [10], and Pd has other advantages, such as low-price, good CO-tolerance, and excellent catalytic activity. Therefore, Pd is probably one of the best candidates for alloying with Pt [11,12].

The size, shape, composition, and surface structure of Pt-based alloy nanoparticles have been shown to significantly influence the catalytic properties. Until today, Pt–Pd bimetallic alloy nanoparticles with all kinds of structures have been fabricated [13,14,15,16], such as cages [2], tetrahedral, dendrites [17], clusters, octahedral [18], and cubes [19]. Compared with their solid counterparts, porous nanoparticles are of great importance due to their larger surface area and lower density; they also exhibit ideal structural features for many catalytic applications. Many different methods [20] have been developed to prepare porous Pt–Pd nanostructures, including galvanic replacement [21], seeded growth [22], solvothermal synthesis [15], co-chemical reduction, and electrochemical deposition [23]. For example, Hong et al. have developed a galvanic replacement route to prepare Pd–Pt alloy nanocages [1] and Pd@Pt core-shell dendritic nanocrystals (NCs) using uniform Pd octahedral and cubic NCs as sacrificial templates [1]. Lim et al. have fabricated Pt–Pd bimetallic nanodendrites, which are composed of a dense array of Pt nano-branches on a Pd core, with uniform Pd nanocrystals being used as crystal-seeds in an aqueous solution [3]. However, the above methods have several disadvantages, including a relatively low cost–performance and multiple reaction steps. Therefore, more cost-effective, novel, and environmentally friendly strategies need to be explored in the synthesis of Pt–Pd bimetallic nanostructures to achieve superior catalytic properties.

Recently, we developed an efficient, one-pot solution-phase strategy to fabricate porous Pt–Pd alloy nanoparticles (Pt–Pd NPs) utilizing a modified polyol process using ethylene glycol (EG) as the reductant, and hydrochloric acid (HCl) and polyvinyl pyrrolidone (PVP) as the structure-directing and stabilizing agents, respectively. No toxic organic solvent, seed, or template was used. These Pt–Pd NPs showed excellent electrocatalytic activity and anti-CO poisoning ability for the oxidation of methanol compared with commercial Pt black (PtB).

## 2. Materials and Methods 

### 2.1. Materials and Reagents

Hexachloroplatinic acid (H_2_PtCl_6_·6H_2_O, ACS reagents), sodium tetrachloropalladate (Na_2_PdCl_4_, 98%), ethylene glycol (EG, >99%) and polyvinyl pyrrolidone (PVP, average *M*_w_ ≈ 55,000) from Sigma-Aldrich (St. Louis, MO, USA); hydrochloric acid (HCl, 37 wt %) and all other solvents (acetone, hexane and ethanol) from SINOPHARM (Shanghai, China) were used for the synthesis of the Pt–Pd nanoparticles. Commercial Pt black (PtB) from RiYn (Shanghai, China) was used as the reference catalysts. All the chemicals were used as received without further purification.

### 2.2. Synthesis of Pt–Pd NPs

The Pt–Pd NPs were synthesized using a modified polyol process. In the process, 2.5 mL ethylene glycol (EG) was refluxed for 5 min at 190 °C in the flask. Then, 0.34 mL of 37 wt % HCl solution was added whilst vigorously stirring. Subsequently, 0.94 mL of PVP 0.0375 M and 0.94 mL of 0.0625 M H_2_PtCl_6_·6H_2_O and Na_2_PdCl_4_ in EG were repeatedly added into the above solution 10 times over a 5 min period. After the addition of the reactants, the solution was again heat-treated for 20 min at 190 °C. The samples were collected using centrifugation and continually washed several times using acetone, ethanol and hexane. Finally, the Pt–Pd NPs were dispersed in an ethanol solution. The reaction temperature, and the ratio of the two precursors (H_2_PtCl_6_/Na_2_PdCl_4_) were changed in order to optimize the reaction conditions.

### 2.3. Structural Characterization

Transmission electron microscope (TEM) and high-resolution TEM (HRTEM) images were taken using a JEOL JEM-2100F microscope operated at the accelerating voltage of 200 kV (Hitachi, Tokyo, Japan). High-angle annular dark-field scanning TEM (HAADF-STEM) images and scanning electron microscopy (SEM) images were obtained using a Magellan 400 microscope operated at the accelerating voltage of 30 kV (FEI, Hillsboro, OR, USA).

Wide-angle and low-angle powder X-ray diffraction (XRD) profiles were obtained with a D8 ADVANCE diffractometer with Cu Kα radiation (Burke, Germany).

### 2.4. Electrochemical Measurements

Cyclic voltammogram (CV) curves were obtained using a CHI 600C electrochemical analyzer (CH Instrument, Shanghai, China). The measurements were conducted using a conventional three-electrode cell in this experiment, where an Ag/AgCl electrode, a platinum wire, and a glassy carbon electrode (GCE, 3 mm in diameter) modified by catalysts were used as the reference electrode, counter electrode, and working electrode, respectively. After being carefully cleaned, the samples were coated on the surface of the GCE with a load of 4 μg. Then, Nafion solution (2.0 μL, 1 wt %) was dropped onto the surface of the samples and was air-dried naturally. The cyclic voltammetry (CV) curves were measured in a 0.1 M H_2_SO_4_ solution saturated by nitrogen at room temperature, in order to determine the electrochemical active surface area (ECSA). Methanol oxidation reaction (MOR) measurements were performed in a 0.5 M H_2_SO_4_ solution containing 1.0 M methanol. The potential scan rate was 50 mV/s for these CV measurements. As a comparison, Pt/C (Alfa) was used as the baseline catalysts, and the electrochemical measurement was performed with the same process as that described above.

## 3. Results

### 3.1. Structural Characterization of Porous Pt–Pd Nanoparticles

**Figure 1:**
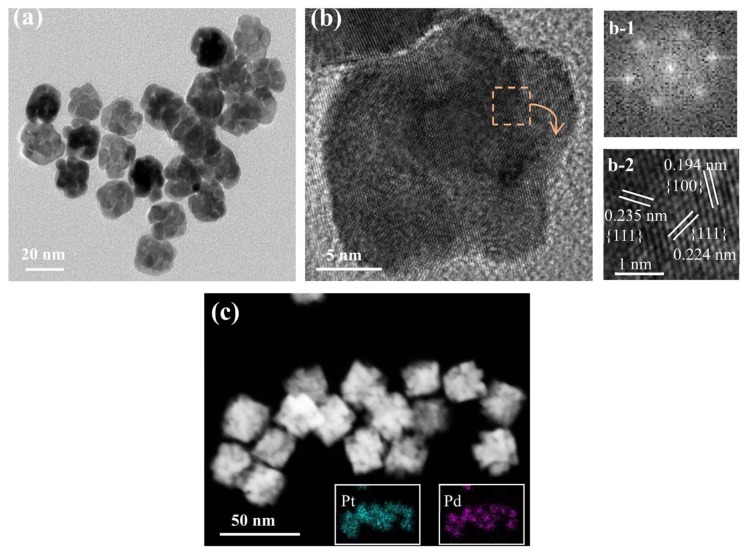
Morphology and composition analyses for the porous Pt–Pd nanoparticles synthesized at 190 °C: (**a**) Representative low-magnification TEM image, (**b**) high-magnification TEM image, (**b-1**) the corresponding Fourier transform (FFT) pattern, (**b-2**) the corresponding magnified TEM image, (**c**) high-angle annular dark-field scanning TEM (HADDF-STEM) image. The insets in (**c**) show the corresponding elemental mapping images of Pt (blue) and Pd (red) for porous Pt–Pd nanoparticles.

**Figure 2:**
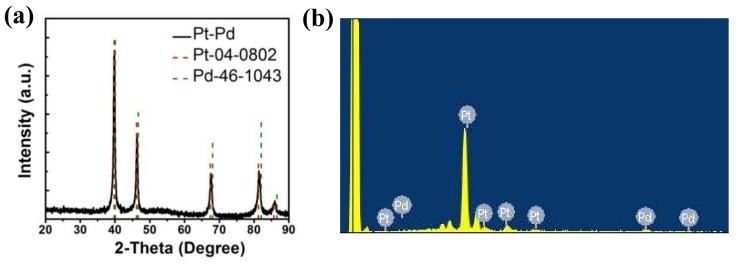
Composition analyses for the porous Pt–Pd nanoparticles synthesized at 190 °C: (**a**) XRD spectrum and (**b**) energy-dispersive spectroscopy (EDS) spectrum.

Figure 1 shows the sizes and morphologies of the synthesized porous Pt–Pd nanoparticles (Pt–Pd NPs) reacted at 190 °C (with a Pt:Pd precursor ratio of 9:1). Well-defined and uniform Pt–Pd NPs with an average size of 19.8 nm were obtained, as shown in Figure 1a. They had a narrow size distribution of 16–25 nm. More importantly, each nanoparticle had a porous structure, with many small particles stacking together, as shown in Figure 1b. From the HRTEM images, the lattice-fringe distances were 0.224 and 0.194 nm (Figure 1b and Figure 1(b-2)), which were close to the (111) and (200) plane of the single Pt (0.2265 nm, 0.1962 nm) and Pd (0.2246 nm, 0.1945 nm), respectively. Its single-crystalline nature could also be elucidated by the corresponding fast Fourier transform (FFT) pattern (inset in Figure 1(b-1)). In addition, XRD measurements were performed to investigate the crystal structures of Pt–Pd NPs. In the XRD spectrum (Figure 2a), these characteristic diffraction peaks of Pt–Pd NPs are located between single Pt (JCPDS-04-0802) and Pd (JCPDS-46-1043), demonstrating the formation of a Pt–Pd bimetallic alloy with a face-centered cubic structure. The ratio of I(111)/I(200) diffraction peak for Pt–Pd NPs in the XRD pattern was obviously larger than that of the Pt nanocrystals with standard crystalline parameters, indicating a higher percentage of (111) crystal planes in Pt–Pd NPs [23]. The STEM image (Figure 1c) of Pt–Pd nanoparticles clearly shows the porous structure with small particles stacking together. The elemental distribution of the Pt–Pd NPs was illustrated in the EDS-mapping images (the insets in Figure 1c). Based on the results, it was revealed that Pt and Pd were homogeneously distributed throughout the nanoparticles. The elemental compositions were investigated using energy-dispersive spectroscopy (EDS) (Figure 2b). The compositional ratio calculated by the EDS spectrum was Pt:Pd = 11:1.

### 3.2. Controllable Synthesis of Pt–Pd NPs

To illustrate the formation process of porous Pt–Pd NPs, we adjusted the reaction conditions, especially the reaction temperature and the ratio of the two precursors, and investigated the influence these adjustments had on the morphologies of the final bimetallic nanoparticles.

**Figure 3:**
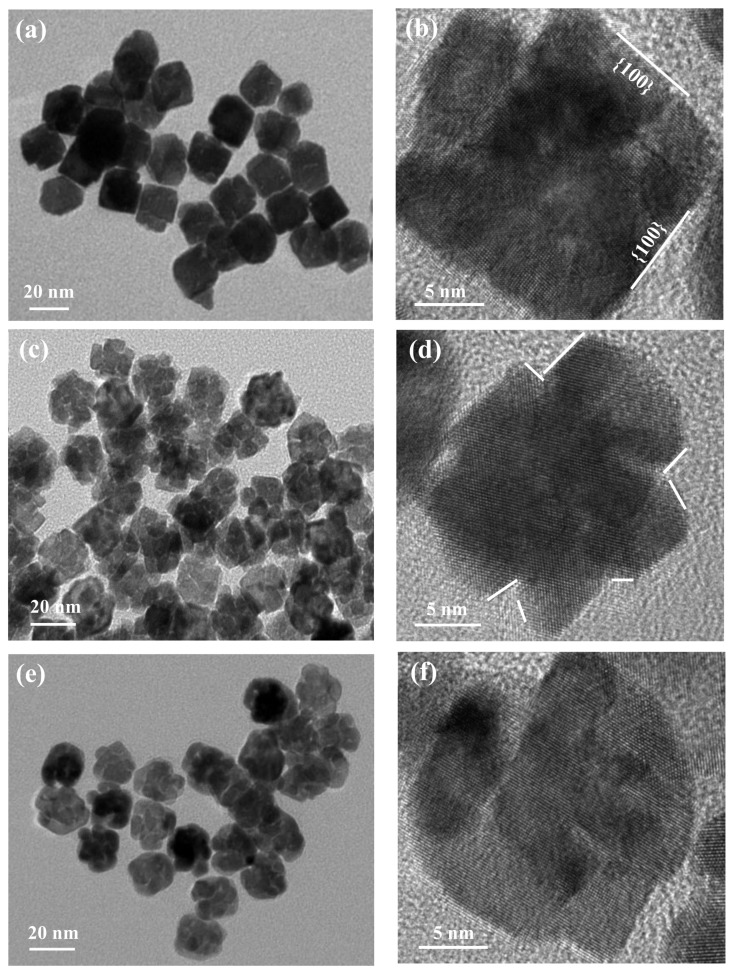
Morphologies of Pt–Pd nanostructures prepared at different temperatures. TEM images: (**a**) 170 °C, (**c**) 180 °C, (**e**) 190 °C; HRTEM images: (**b**) 170 °C, (**d**) 180 °C, (**f**) 190 °C.

As demonstrated in various studies, the temperature is one of the most important parameters affecting the decomposition and reduction rate of the metal precursor. This acts to change the growth kinetics by adjusting the equilibrium established between the different species found in the reaction solution. As shown in Figure 3a,b, cubic nanoparticles were formed without the stacking phenomenon when the temperature was 170 °C. However, the porous alloy structures stacked at 180 °C (Figure 3c) and 190 °C (Figure 3e), respectively. In addition, there are some lattice fringes observed on the surfaces, all of which are beneficial to achieving a high catalytic activity. It can be inferred from this that more crystal nuclei generate at higher temperatures, and thus grow into particle-stacking structures, which would result in a higher surface area and more active sites conducive to better catalytic properties [4]. Additionally, from the HRTEM images in Figure 3b,d,f, no defects can be seen in the stacking structures which demonstrates that these alloy particles are of single-crystalline structures.

**Figure 4:**
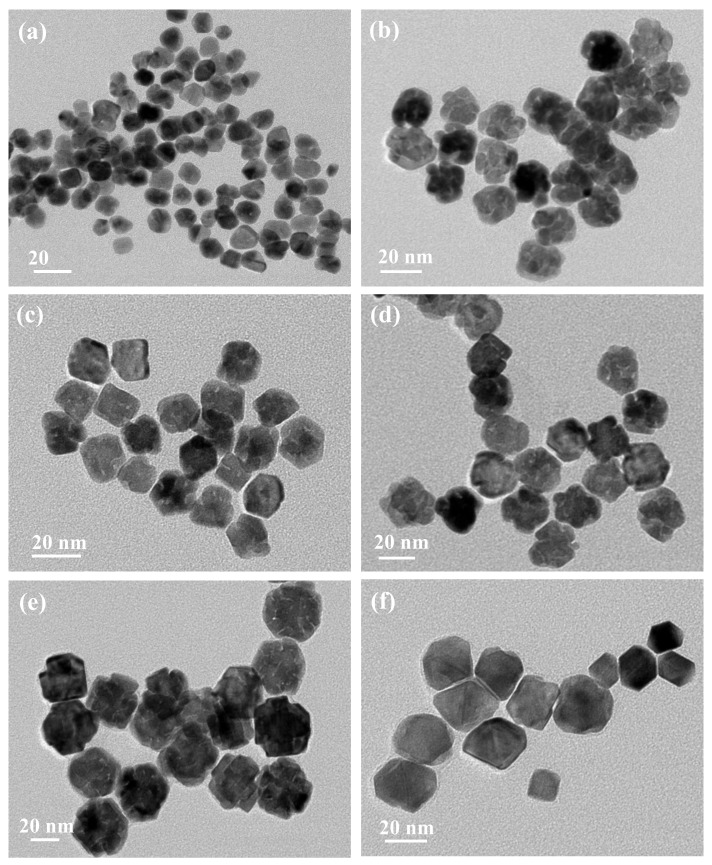
TEM images of Pt–Pd nanoparticles prepared by adding precursors with different ratios: (**a**) 10:1, (**b**) 9:1, (**c**) 7:1, (**d**) 5:1, (**e**) 3:1, (**f**) 1:1.

In addition to temperature, the ratio of the two different precursors is another important factor in adjusting the rate of reaction and thus the morphologies of the final nanostructures. In this study, the molar ratio of H_2_PtCl_6_·6H_2_O and Na_2_PdCl_4_ were adjusted between a range of 1~10. As shown in Figure 4, the size of the Pt–Pd bimetallic nanoparticles increased from 12, 19, 20, 22, to around 30 nm with the decrease in the Pt/Pd precursor ratio. When the ratio is 10 (Figure 4a) or 1 (Figure 4f), small nanoparticles were obtained without a stacking structure. When the ratio was 9 (Figure 4b), 7 (Figure 4c), 5 (Figure 4d), and 3 (Figure 4e), nanostructures with small particles stacking together were obtained; this illustrates that the stacking structures could be obtained under the appropriate ratio. The porous structure with stacking particles is most notable when the ratio is 9. The special structure with small particles stacking together is helpful in improving the catalytic activity. As we know, the reduction potential of PdCl^4−^/Pd^0^ (0.62 V vs. RHE) is smaller than that of PtCl^4−^/Pt^0^ (0.758 V vs. RHE). Therefore, some Pd^4+^ ions in this solution may be easily deoxidized and serve as deposition or nucleation sites. As the Pt/Pd precursor ratio decreases, the amount of Pd nuclei increases, leading to an increase in the formation of the stacking-particle structure (Figure 4b). With a continual decrease in the Pt/Pd precursor ratio, the growth of Pd nuclei is in a dominant position, and thus leads to the increase of alloy particles’ size (Figure 4e,f), not the formation of more stacking-particles structures.

### 3.3. Electrocatalytic Properties

In order to characterize the catalytic properties of the Pt–Pd nanoparticles obtained at different temperatures and different ratios of precursors, the electrochemical properties of Pt–Pd nanoparticles were analyzed using a three-compartment electrochemical cell.

**Figure 5:**
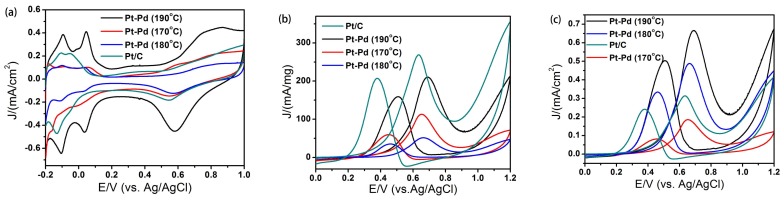
Cyclic voltammetry (CV) curves of Pt–Pd nanoparticles prepared at different temperatures and commercial Pt/C: (**a**) in 0.5 M H_2_SO_4_ solution; (**b**) in 1.0 M methanol + 0.5 M H_2_SO_4_ solution, mass activities; and (**c**) in 1.0 M methanol + 0.5 M H_2_SO_4_ solution, specific activities. The scan rate was 50 mV/s.

Figure 5 shows the cyclic voltammetry (CV) curves of Pt–Pd nanoparticles obtained at different temperatures. The CV curves in Figure 5a were recorded at room temperature in 0.1 M H_2_SO_4_ solutions. These curves exhibit two distinctive potential regions associated with the H_upd_ adsorption/desorption process and the formation of an OH_ad_ layer, which are typical Pt-like H_upd_ features. On the basis of the CV data, the electrochemically active surface area (ECSA) of catalysts was calculated by measuring the charge collected in the H_upd_ adsorption/desorption region. In addition, the electrocatalytic behaviors were examined in a methanol oxidation reaction system. Figure 5b,c shows the mass and specific activities of catalysts for the methanol oxidation reaction. The catalysts showed typical features found in the oxidation process of methanol, which contain characteristic double anodic peaks in the forward and reverse scans. The calculated results are shown in Table 1. The area specific activity of Pt–Pd NPs obtained at 190 °C, 180 °C, 170 °C and Pt/C was 0.67, 0.49, 0.19 and 0.31 mA·cm^−2^, respectively. This result demonstrates that nanoparticles obtained at 190 °C show the highest catalytic activity, and the specific activity is about 2.13 times that of the commercial Pt/C catalyst. The increased activity of Pt–Pd NPs could be attributed to their unique structures with the porous features and a higher percentage of Pt {111} planes exposed [23]. Firstly, the Pt–Pd NPs exhibit a three-dimensional interconnected porous structure with a large surface area and an interconnected network composed of nano-branches; this is helpful for electron and mass transfer in catalytic reactions. Secondly, it is possible for the electronic, strain, or alloy effects to take place in the unique hierarchical Pt–Pd bimetallic structure, resulting in a further enhanced catalytic reactivity [24]. 

**Figure 6:**
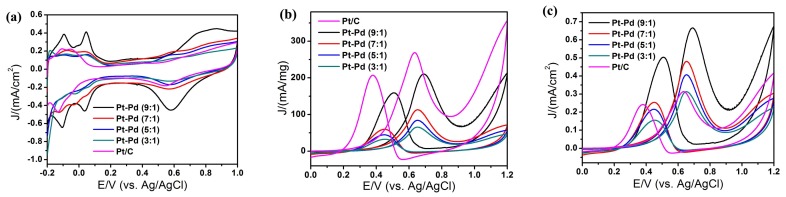
CV curves of Pt–Pd nanoparticles prepared by adding precursors with different ratios and commercial Pt/C: (**a**) in 0.5 M H_2_SO_4_ solution; (**b**) in 1.0 M methanol + 0.5 M H_2_SO_4_ solution, mass activities; (**c**) in 1.0 M methanol + 0.5 M H_2_SO_4_ solution, specific activities. The scan rate was 50 mV/s.

Figure 6 shows the cyclic voltammetry (CV) curves of Pt–Pd nanoparticles obtained at different ratios of precursors. From Figure 6a, we calculated the ECSA of the catalysts by measuring the charge collected in the H_upd_ adsorption/desorption region. In addition, the electrocatalytic behaviors were examined in a methanol oxidation reaction system. Figure 6b,c shows the mass activities and specific activities of catalysts for the methanol oxidation reaction; characteristic double anodic peaks in the forward and reverse scans were observed, indicating a typical feature of the oxidation process of methanol. The results are summarized in Table 2, and it indicates that the catalytic activity decreases with the decrease of the ratio of Pt/Pd, while the ratio (I_f_/I_b_) of the forward current density (I_f_) to the backward current density (I_b_) increases with the decrease of the ratio of Pt/Pd. This demonstrates that it is beneficial to improve the anti-CO poisoning performance.

## 4. Conclusions

In summary, this study presents an efficient, one-pot route for the synthesis of porous Pt–Pd bimetallic nanostructures without using seeds, templates and toxic reactants. The Pt–Pd NPs had a porous feature and single-crystalline nature, with small particles stacking together. Thus, they exhibited an excellent catalytic activity for the oxidation of methanol with a higher current density (0.67 mA/cm^2^) than the commercial Pt/C catalyst (0.31 mA/cm^2^). In addition, it was found that the anti-CO poisoning properties improved as the amount of palladium increased. Our method provides a possibility for the easy and economical preparation of different alloy nanostructures for various applications.

## Figures and Tables

**Table 1:** The catalytic activities of Pt–Pd nanoparticles at different temperatures and commercial Pt/C for methanol oxidation reaction.

Catalysts	ECSA (m^2^/g)	Mass Activity (mA/mg)	Specific Activity (mA/cm^2^)
Pt–Pd-190 °C	31.59	210.35	0.67
Pt–Pd-180 °C	10.54	51.48	0.49
Pt–Pd-170 °C	35.48	65.85	0.19
Pt/C	85.63	268.3	0.31

**Table 2:** The catalytic properties of Pt–Pd nanoparticles prepared by adding precursors with different ratios and commercial Pt/C for methanol oxidation reaction.

Catalysts	ECSA (m^2^/g)	Mass Activity (mA/mg)	Specific Activity (mA/cm^2^)	I_f_/I_b_
Pt–Pd (9:1)	31.59	210.35	0.67	1.32
Pt–Pd (7:1)	23.51	112.95	0.48	1.89
Pt–Pd (5:1)	20.70	84.20	0.41	1.89
Pt–Pd (3:1)	20.90	65.60	0.31	2.02
Pt/C	85.63	268.3	0.31	1.29
